# Longitudinal research on the dynamics and internal mechanism of female entrepreneurs’ passion

**DOI:** 10.3389/fpsyg.2022.1037974

**Published:** 2022-11-04

**Authors:** Xiaorong Fu, Yaling Ran, Qian Xu, Tianshu Chu

**Affiliations:** ^1^School of Business Administration, Southwestern University of Finance and Economics, Chengdu, Sichuan, China; ^2^School of Business Administration, Hong Kong Baptist University, Hong Kong, Hong Kong SAR, China

**Keywords:** entrepreneurial passion, female entrepreneurs, entrepreneurial effort, fear of failure, latent growth model, cross-lag model

## Abstract

Based on Vallerand’s dualistic model of passion, this study theorizes and empirically examines the temporal dynamics of two types of entrepreneurial passion in female entrepreneurs, harmonious entrepreneurial passion (HmEP) and obsessive entrepreneurial passion (ObEP), and examines the mechanisms by which entrepreneurial effort0 and fear of failure influence the temporal dynamics of entrepreneurial passion. Using data collected from a three-wave, lagged survey of female entrepreneurs, we employed Mplus to build a latent growth model for entrepreneurial passion and built a cross-lag model of the relationship between entrepreneurial passion, entrepreneurial effort, and fear of failure. We found that female entrepreneurs’ HmEP and ObEP present different temporal dynamics. Furthermore, the temporal dynamics of HmEP are achieved through changes in entrepreneurial effort, whereas the temporal dynamics of ObEP are achieved through changes in current entrepreneurial effort and fear of failure in the next stage. Therefore, due to traditional gender stereotypes and varying motivations to engage in entrepreneurship, the two entrepreneurial passions have different dynamic evolution processes. Our results underscore the importance of effort and fear of failure in stimulating the dynamics of female entrepreneurial passion.

## Introduction

Entrepreneurship improves women’s quality of life and elevates their social status (Datta and Gailey, 2012). It also contributes to overall economic growth and social well-being (Mari et al., 2016; Xie and Wu, 2021). According to the 2017–2018 Global Entrepreneurship Monitor Report released by the Global Entrepreneurship Monitor (GEM), among a group of 50 countries that participated in the same survey in 2016 and 2017, the rate of female entrepreneurship increased by 6.6%, while male entrepreneurship rose by only 0.7%. Alibaba’s 2019 Global Women’s Entrepreneurship and Employment Research Report noted that the golden age of female entrepreneurship is beginning. Nevertheless, women are less involved in entrepreneurial activities remains than men (Shinnar et al., 2012; Thébaud, 2015; Xie and Wu, 2021). Moreover, female entrepreneurs are more likely than males to abandon entrepreneurship voluntarily (Justo et al., 2015), indicating a lack of stability in their entrepreneurial investments.

Entrepreneurship has traditionally been viewed as a male vocation and has been associated with masculinity (Bird and Brush, 2002; Malmström et al., 2017). Traditional gender roles hold that a woman’s place is in the home and a man’s place is in the workplace (Eagly and Karau, 2002). As a result, women tend to evaluate their professional abilities and options more negatively than men (Dempsey and Jennings, 2014) and have lower entrepreneurial aspirations and intentions (Gupta et al., 2008, 2009; Ward et al., 2019). Furthermore, their entrepreneurial practices are more influenced by family or domestic factors (Jennings and McDougald, 2007; Hsu et al., 2016), and this often limits their entrepreneurial passion (Murnieks et al., 2020). It should be noted that there is embedded gender bias in existing empirical research on entrepreneurial passion in that male samples are considerably larger than female samples (de Mol et al., 2016; Murnieks et al., 2020; Kiani et al., 2021). In some cases, male samples are twice as large as female samples (Stroe et al., 2019; de Mol et al., 2020; Luu and Nguyen, 2020; Su et al., 2022), making traditional research conclusions on entrepreneurial passion highly skewed to male entrepreneurs.

Entrepreneurial passion is a key driver of entrepreneurial innovation (Luu and Nguyen, 2020). The steady development of entrepreneurial passion is critical for entrepreneurs (Murnieks et al., 2014; Clarysse et al., 2015; Xiao and Fu, 2022), entrepreneurial enterprises (Cardon et al., 2009; Adomako and Ahsan, 2022), and investors and other stakeholders (Breugst et al., 2012; Warnick et al., 2018). Vallerand (2015) argues that the development of passion is an ongoing process and the level and type of passion one has may change or fluctuate over time. Although psychologists have conducted longitudinal studies on individual passion to explore dynamic development trends (Tóth-Király et al., 2018; Kovácsik et al., 2020), scholars have largely ignored the dynamic evolution of entrepreneurial passion, and most of the research using cross-sectional data does not align with the reality of the dynamic development of entrepreneurial passion (de Mol et al., 2016; Türk et al., 2019; Murnieks et al., 2020). Considering that entrepreneurship is an ongoing, dynamic development process (Lichtenstein et al., 2007), and individual passion itself is a variable that fluctuates over time, understandings about entrepreneurial passion based on a static perspective have limited ability to explain the success or failure of entrepreneurial endeavors. Therefore, many scholars in the field have called for longitudinal studies of entrepreneurial passion (Ho and Pollack, 2014; de Mol et al., 2016; Türk et al., 2019; Kiani et al., 2021). However, to date, only a few studies have examined changes in entrepreneurial passion over time from a dynamic perspective (Cardon et al., 2013; Gielnik et al., 2015; Collewaert et al., 2016; Lex et al., 2020; Uy et al., 2021), and those studies that exist have largely ignored gender differences or focused mainly on male entrepreneurs.

With respect to female entrepreneurship, what entrepreneurial passion development trends present? What factors influence female entrepreneurial passion development trends? In order to examine these issues in depth, first, we conducted a cross-temporal study on the entrepreneurial passion of female entrepreneurs based on a dualistic model of passion by building a latent growth model that assessed development trends of female entrepreneurs’ harmonious entrepreneurial passion (HmEP) and obsessive entrepreneurial passion (ObEP). We then explored the factors that affected changes in the entrepreneurial passion of the female entrepreneurs by building a cross-lag model. This enabled us to examine and describe in depth the dynamic evolution of female entrepreneurs’ entrepreneurial passion over time, discover process differences of the dynamic evolution of female entrepreneurs’ HmEP and ObEP, and enhance theoretical support for better management of and support for women’s entrepreneurial practices.

Our study offers the following contributions. First, unlike most extant literature on entrepreneurial management, we focused specifically on female entrepreneurs and found that the entrepreneurial passion of female entrepreneurs is indeed dynamic.

Most existing entrepreneurship research regard entrepreneurial passion as a stable, constant characteristic (Baum et al., 2001; Baum and Locke, 2004) and, based on this assumption, study the impact of entrepreneurial passion on entrepreneurial intention (Biraglia and Kadile, 2017), corporate innovation (Luu and Nguyen, 2020), and corporate performance (Su et al., 2022). But this precept has been challenged in recent years. Cardon et al. (2012) suggest collecting data at multiple time points and investigating the non-static nature of passion, and Vallerand (2015) posits that the amount and type of passion an individual possesses may change or fluctuate over time. However, to our knowledge, only a few extant studies have examined the evolution of entrepreneurial passion from a dynamic perspective (Cardon et al., 2013; Gielnik et al., 2015; Collewaert et al., 2016; Lex et al., 2020; Uy et al., 2021). Most of these studies are based on the entrepreneurial passion model of Cardon et al. (2009), and none of them distinguishes the genders of entrepreneurs.

Researchers have previously noted gender differences in entrepreneurship (Eddleston and Powell, 2012; Murnieks et al., 2020). Due to gender stereotypes and social exigencies (Ahl and Marlow, 2012), the entrepreneurial opportunities (DeTienne and Chandler, 2007) and barriers (Bates, 2002; Shinnar et al., 2012; Cardella et al., 2020) female entrepreneurs encounter are quite different from those of male entrepreneurs. Women entrepreneurs are more influenced by family and/or domestic factors (Shelton, 2006; Eddleston and Powell, 2012; Phipps and Prieto, 2014; De Clercq and Brieger, 2021). As a result, the passion and effort of female entrepreneurs throughout the entrepreneurial process is more volatile (Digan et al., 2019; Murnieks et al., 2020). Thus, while it is important to hone in on this dynamic among female entrepreneurs, existing research have largely overlooked this cohort as indicated by the skewed gender ratios in empirical research samples on entrepreneurial passion. It is notable that male samples are generally much larger than female samples (de Mol et al., 2016, 2020; Stroe et al., 2019; Luu and Nguyen, 2020; Murnieks et al., 2020; Kiani et al., 2021; Su et al., 2022), and research conclusions are more connoted to male entrepreneurs. This study uses follow-up survey data of 88 Chinese female entrepreneurs at three points in time over 5 months, focuses on dynamic changes in these women’s entrepreneurial passion, and adds a unique perspective to the body of research on female entrepreneurship.

Secondly, based on Vallerand’s dualistic model of passion (DMP), we endeavor to reveal the dynamic change processes of HmEP and ObEP of female entrepreneurs and found that the dynamic change trends of their HmEP and ObEP do differ. Some scholars hold that women participate in entrepreneurship activities either for (a) internal factors such as the pursuit of opportunity and independence (Walker and Webster, 2007; Jafari-Sadeghi, 2020), or (b) external factors such as involuntary unemployment or a desire to be recognized (Ghatak and Bhowmick, 2021; Jafari-Sadeghi et al., 2021). We found that these factors cannot be fully reflected in the entrepreneurial passion model proposed by Cardon et al. (2009). Therefore, we employed Vallerand’s DMP as a way to describe the dynamic evolution of female entrepreneurial passion, using HmEP and ObEP to reflect the entrepreneurial motivation of female entrepreneurs. We found that female entrepreneurs who experience HmEP deal with conflicts between work and family domains in a harmonious way, thus keeping HmEP at a reasonable level; and female entrepreneurs with high ObEP focus more on the entrepreneurial role, thereby continuously increasing ObEP for entrepreneurial activities, which provides new theoretical insights into the dynamics of entrepreneurial passion.

Finally, we considered the differing impacts of female entrepreneurs’ fear of failure and entrepreneurial effort in the dynamic evolution of HmEP and ObEP, and found that female entrepreneurs’ entrepreneurial effort has a stimulating effect on subsequent changes of HmEP, while entrepreneurial effort has a stimulating effect on subsequent changes of ObEP by affecting fear of failure. Due to the influence of gender stereotypes, female entrepreneurs evince a higher fear of failure than male entrepreneurs (Koellinger et al., 2013), and there is greater volatility in their degree of attention to their entrepreneurial endeavors (McGowan et al., 2012; Digan et al., 2019). Extant studies have identified entrepreneurial passion as a factor driving entrepreneurial effort (Baum and Locke, 2004; Cardon et al., 2009)，and entrepreneurial effort predicts an increase in positive emotions in subsequent entrepreneurial passion (Gielnik et al., 2015). Thus，when female entrepreneurs experience higher HmEP or higher ObEP, they put more effort into their entrepreneurial activities, and the effort causes subsequent changes in HmEP and ObEP. Furthermore, since female entrepreneurs who experience HmEP often participate in entrepreneurship because of inner motivation to pursue opportunities and independence, they can autonomously control their entrepreneurial activities and are less susceptible to failure syndrome (LeRouge et al., 2006). However, because female entrepreneurs who have ObEP are often forced to start a business due to involuntary unemployment or the pursuit of recognition by others, and they are controlled by their entrepreneurial activities (Vallerand et al., 2003; Vallerand, 2015), and they are overly sensitive to the threat of failure (Fox et al., 2002). Therefore, our study incorporates fear of failure and entrepreneurial effort into the research framework in an effort to discover their differing roles in the dynamic evolution of HmEP and ObEP. Using a cross-lag model, the stimulating roles of the two variables in the evolution of HmEP and ObEP are verified, and the internal driving mechanism of the evolution of HmEP and ObEP is described and interpreted. This study expands the body of knowledge on the dynamics of entrepreneurial passion and offers novel research constructs on the entrepreneurial passion of female entrepreneurs.

## Theoretical background and hypotheses

### Female entrepreneurship

Researchers have begun to focus on gender differences in the entrepreneurial environment (Ahl and Marlow, 2012), as well as entrepreneurial opportunities (DeTienne and Chandler, 2007), entrepreneurial barriers (Bates, 2002; Shinnar et al., 2012; Wilson, 2015; Cardella et al., 2020), and entrepreneurial action(Botha, 2020). Due to gender stereotypes and various other factors, the entrepreneurial motives of female entrepreneurs are different from those of their male counterparts, and these differences lead to different entrepreneurial outcomes.

Some women engage in entrepreneurship in pursuit of independence. Yet women often experience significant role conflict between work and family (Frone et al., 1992; Ufuk and Özgen, 2001; Noor, 2004; Welter, 2004; Hsu et al., 2016; De Clercq and Brieger, 2021) resulting in a negative impact on their well-being (Hammer et al., 2004) and entrepreneurial performance (Shelton, 2006). Working women are often challenged to find an appropriate balance between the demands of work and family (O’Neil et al., 2008; Phipps and Prieto, 2014). The balance between the work and family will improve their work motivation, job satisfaction, and business performance (Shelton, 2006; Beutell, 2012; Welsh et al., 2017). Balancing family and work roles is, therefore, a high priority for female entrepreneurs (De Clercq and Brieger, 2021). Entrepreneurship offers women an opportunity to earn income while also providing independence and the flexibility (Jafari-Sadeghi, 2020) to apportion their time and energy to various life domains. The ability to balance family and work demands often stimulates women’s intrinsic motivation to participate in entrepreneurship.

However, some women are forced to start businesses out of necessity. The dominant culture in many countries serves male stereotypes, and women have limited access to financial and human resources (Alonso-Almeida, 2013; Cozarenco and Szafarz, 2015). As a result, factors such as insufficient household income, unemployment, and job dissatisfaction can drive women’s participation in entrepreneurship. Alternatively, women may launch businesses to gain recognition (Ghatak and Bhowmick, 2021). These women may feel that their achievements in the corporate milieu are undervalued, that they cannot achieve leadership positions (Atwater et al., 2001; Cortina et al., 2011) or receive due recognition for their accomplishments (Cortina et al., 2011). Because women are underrepresented among entrepreneurs, successful female entrepreneurs attract attention and gain recognition (Patterson and Mavin, 2009). Entrepreneurs-by-necessity are less willing to take risks than opportunistic entrepreneurs (Block et al., 2015). Being generally risk-sensitive, women are more likely to engage in entrepreneurship due to extrinsic motivations such as involuntary unemployment or the pursuit of recognition.

### Research on the concept and dynamics of entrepreneurial passion

Scholars have employed three theoretical models to study the concept of entrepreneurial passion (Zhao and Liu, 2022). The first model, developed by Baum and Locke (2004), is about general work passion and posits that passion is a specific emotional experience, such as love, enthusiasm, joy, or desire, and that people with passion work hard to maintain that positive feeling. The second is a role-based entrepreneurial passion model developed by Cardon et al. (2009, 2013) which defines entrepreneurial passion as “consciously accessible intense positive feelings experienced by engagement in entrepreneurial activities associated with roles that are meaningful and salient to the self-identity of the entrepreneur.” These scholars focus on entrepreneurial passion in three domains of entrepreneurship, namely inventing, founding, and developing. The third is the dualistic passion model (DPM) developed by Vallerand et al. (2003) based on self-determination theory (Deci and Ryan, 2000), in which passion is defined as “a strong inclination toward an activity that people like, that they find important, and in which they invest time and energy.” The model distinguishes between harmonious passion and obsessive passion. *Harmonious passion* refers to an autonomous internalization that guides individuals to choose to engage in activities they prefer. *Obsessive passion* is the controlled internalization of an activity as a person, creating internal pressure to engage in an activity that the person enjoys. The DPM was originally developed for hobbies (e.g., gambling), and in recent years scholars have applied it to the entrepreneurial context.

In this study, we explore the entrepreneurial passion of female entrepreneurs based on the model of Vallerand et al. (2003) for three reasons. First, we are interested in female entrepreneurs who either actively participate in entrepreneurial activities due to independent intrinsic motivation or are forced to start a business due to extrinsic motivations such as involuntary unemployment or the pursuit of recognition by others. The DPM is divided into harmonious passion and obsessive passion according to how activities are internalized into an individual’s core identity. Individuals with harmonious passion can control their activities while those with obsessive passion are controlled by activities. We posit that this model best describes the entrepreneurial passion of female entrepreneurs generated by different motivations. Second, we are interested in general entrepreneurial passion, not the passion for a specific entrepreneurial domain. Extant research has shown that the DPM is suitable for conceptualizing entrepreneurial passion broadly (Ho and Pollack, 2014; Murnieks et al., 2016; Gielnik et al., 2017). Third, we are interested in the dynamics of entrepreneurial passion, and the DPM details the internalization of passion over time (Mageau et al., 2009).

Therefore, we draw on the DPM to define entrepreneurial passion, reasoning that entrepreneurial passion is a strong inclination towards entrepreneurial activities that people enjoy, that they consider important, and in which they invest time and energy. Entrepreneurial passion may be harmonious entrepreneurial passion (HmEP) or obsessive entrepreneurial passion (ObEP). Although these are both positive emotions in the entrepreneurial context, the generation of the two passions and their impacts on entrepreneurs differ significantly (Stroe et al., 2018a). HmEP stems from positive expectations about the anticipated value of entrepreneurial activities (Vallerand et al., 2003; Vallerand, 2015). Among entrepreneurs who experience HmEP, entrepreneurial activities become an integral part of their self-concept, occupying an important although not overwhelming position. The entrepreneurial identity and other aspects of the individual’s identity (e.g., vis-à-vis spouse, parents, etc.) coexist harmoniously. Thus, the entrepreneur does not feel undue external pressure (e.g., about self-worth and recognition) and is able to maintain autonomy in his/her entrepreneurial activities (Vallerand, 2015). By contrast, ObEP emerges from intrapersonal or interpersonal pressures attached to entrepreneurial activities, such as the expectation of social recognition, self-esteem, and/or the need to reduce uncertainty (Vallerand et al., 2003; Vallerand, 2015). These pressures tend to dominate entrepreneurs and compel them to continue investing in entrepreneurial activities (Stroe et al., 2018b). This means that entrepreneurs view engagement in entrepreneurial activities as important because they aspire to achieve certain results related to the activities or to satisfy social needs (e.g., social acceptance, self-worth, and superiority). Entrepreneurs who experience ObEP are controlled by their entrepreneurial activities and often cannot independently control their participation in them. Entrepreneurship occupies a dominant position in the identity of such individuals and may conflict with other identities in their lives.

Extant research on entrepreneurial passion use mostly cross-sectional studies (de Mol et al., 2016; Türk et al., 2019; Murnieks et al., 2020). However, since entrepreneurship is a continuous and dynamic process (Lichtenstein et al., 2007), the conclusions drawn from research on entrepreneurial passion based on a static perspective have limited explanatory power. Thus, longitudinal research on entrepreneurial passion is very important (Türk et al., 2019; Boone et al., 2020). To our knowledge, only a few studies have examined how entrepreneurial passion evolves from a dynamic perspective (Cardon et al., 2013; Gielnik et al., 2015; Collewaert et al., 2016; Lex et al., 2020; Uy et al., 2021). And while these studies confirm that entrepreneurial passion is dynamic over time, they do not differentiate between genders. Traditional gender stereotypes hold that the proper place for women is at home while men are at work (Eagly and Karau, 2002) and give rise to the view that there is a disconnect between “women” and “entrepreneurship.” To the best of our knowledge, most previous studies on entrepreneurial passion view gender as a control variable, with only one study considering gender as a moderating variable (Murnieks et al., 2020). Since no detailed study of passion in female entrepreneurs exists, we set out to explore the dynamics of female entrepreneurs’ HmEP and ObEP from a dynamic perspective.

### The dynamics of female entrepreneurs’ entrepreneurial passion

Considering the differences between HmEP and ObEP, we speculate that female entrepreneurs who experience HmEP deal with conflicts between work and family domains in a harmonious way, thus keeping HmEP at a reasonable level.

Individuals who experience harmonious passion are able to autonomously manage their activities such that each role comprises an important but not overwhelming part of their identity (Vallerand et al., 2003; Vallerand, 2015). These entrepreneurs value their multiple role identities and provide each role identity with appropriate resources (Burke and Reitzes, 1991; Clark, 2000). As a result, female entrepreneurs who have HmEP can autonomously decide if and when to allocate resources and engage in various activities (Hobfoll, 2001).

The entrepreneurial role and family role are critical roles for female entrepreneurs (Shelton, 2006; Zhu et al., 2021). Prior studies have found that when women have autonomy at work, they have flexibility to direct resources to balance work and family demands (Quinn et al., 2012; Halliday et al., 2018), and are satisfied with their ability to cope with the dual demands (De Clercq and Brieger, 2021). Thus, we speculate that female entrepreneurs who experience HmEP are able to allocate appropriate resources to both of these roles and adequately maintain work-family balance.

“Work-family balance” is defined as “a state of equilibrium in which an individual’s work and family needs are equal” (Duxbury and Higgins, 2001). However, based on the perspective of resource scarcity, an individual’s personal resources (e.g., time, attention, and energy) are limited, and meeting the needs of one role inevitably limits the ability to meet the needs of other roles (Hammer et al., 2009). Therefore, we posit that female entrepreneurs who experience high HmEP in the early stages will gradually reduce their entrepreneurial passion over time in order to balance their multiple roles. By contrast, female entrepreneurs with low HmEP in the early stages will devote more passion to entrepreneurial activities, so HmEP will rise. Thus, we propose the following hypothesis:

*H1a*: HmEP of female entrepreneurs shows a trend of heterogeneity over time. A subgroup whose initial HmEP is high will present a downward trend of HmEP over time. A subgroup whose initial HmEP is low will present an upward trend.

Unlike those with HmEP, we speculate that female entrepreneurs with high ObEP focus more on the entrepreneurial role, thereby continuously increasing ObEP for entrepreneurial activities.

Obsessive passion is the result of controlled internalization of an activity into one’s identity (Vallerand et al., 2003; Vallerand, 2015). Esther Alonso-Galicia et al. (2015) found that, when deciding to start a business, expected social support has a greater impact on the entrepreneurial intentions of females than males. This suggests that once women internalize their entrepreneurial role identity and realize that this identity leads to good outcomes, they are compelled to increase their passion for entrepreneurship.

However, individuals with obsessive passion internalize entrepreneurial activity as a result of personal and/or interpersonal pressures either because some contingency, such as social acceptance or self-esteem, is attached to the activity or because they are drawn by the lure of activity-generated excitement (Vallerand et al., 2003; Vallerand, 2015). Yet women entrepreneurs are more likely to fail due to lack of access to funding (Fairlie and Robb, 2009), lack of management and administrative experience (Boden and Nucci, 2000; DeTienne and Chandler, 2007), and/or lack of a viable business strategy (Carter et al., 1997). Female entrepreneurs who experience high ObEP are unable to control their entrepreneurial activities independently and feel heavy responsibility for the entrepreneurial role. Accordingly, they allocate a great deal of energy and time to this role (Ashforth and Mael, 1989; Burke and Reitzes, 1991; Stryker and Serpe, 1994). In the case of high ObEP, they are likely to continue to push the entrepreneurial role boundary (Ashforth et al., 2000) and regard success in this role as the realization of their self-worth (Burke and Reitzes, 1991; Clark, 2000).

Based on this research, we speculate that female entrepreneurs who have high ObEP are more likely to identify with the entrepreneurial role. And because participation in entrepreneurial activities is out of their control given the male dominated social and economic environment, they will allocate more energy and time to the entrepreneurial role until it eventually engulfs them. As a result, their ObEP becomes higher and higher over time.

Conversely, female entrepreneurs who exhibit low ObEP at early stages consider the entrepreneurial role less important, and they are less controlled by entrepreneurial activities. We speculate that low importance and lack of control over the entrepreneurial role may further diminish their passion for entrepreneurial activities. Moreover, many female entrepreneurs attach great importance to the quality of life associated with business success (Cliff, 1998). As a result, they may succumb to personal distractions (Brush, 1992), temper their growth aspirations, and pursue non-economic goals (Cliff, 1998). Consequently, female entrepreneurs are more likely than males to abandon entrepreneurship for personal reasons (Justo et al., 2015). However, female entrepreneurs who have low ObEP also have limited resources to invest in entrepreneurial activities, and so they may not achieve their entrepreneurial goals. In such cases, these female entrepreneurs may opt out of entrepreneurial projects because they have failed to gain self-esteem or social recognition. Therefore, over time, female entrepreneurs who have low ObEP at early stages are more likely to relinquish control of entrepreneurial activities, and their ObEP shows a downward trend. We seek to confirm this relationship by hypothesizing:

*H1b*: ObEP of female entrepreneurs shows a trend of heterogeneity over time. A subgroup whose initial ObEP is high will present an upward trend of ObEP over time, A subgroup whose initial ObEP is low will present a downward trend.

### Mediating role of entrepreneurial effort

Passion is a positive emotion (Watson, 1988), and Foo et al. (2009) found that entrepreneurs’ positive emotions boost their level of entrepreneurial effort. Entrepreneurial effort is the energy that entrepreneurs put into the process of starting and running a business, including not only time and material resources but also spiritual and emotional outlays (Gielnik et al., 2017). Extant studies have identified entrepreneurial passion as a factor driving entrepreneurial effort (Baum and Locke, 2004; Cardon et al., 2009). Furthermore, the self-validation literature suggests that identity guides the behavior of individuals as it motivates them to reaffirm the existence of the behavior (Burke and Stets, 1999). Since HmEP and ObEP are the results of autonomous and forced internalization of entrepreneurial activities into identity, entrepreneurial identity will prompt female entrepreneurs to work harder.

Furthermore, according to control theory, increased effort enables individuals to make progress toward their goals, which further stimulates positive emotions (Carver and Scheier, 1982; Carver, 2006). A longitudinal study has shown that entrepreneurial effort predicts an increase in positive emotions in subsequent entrepreneurial passion (Gielnik et al., 2015). Therefore, we believe that the entrepreneurial effort of female entrepreneurs plays a mediating role in the dynamics of HmEP and ObEP. In other words, when female entrepreneurs experience higher HmEP or higher ObEP, they put more effort into their entrepreneurial activities, and the effort causes subsequent changes in HmEP and ObEP. Therefore, we propose Hypothesis 2.

*H2*: Entrepreneurial effort play a positive mediating role in the dynamics of (a) HmEP and (b) ObEP of female entrepreneurs.

### Mediating role of fear of failure

In the entrepreneurship context, fear of failure is a negative emotion generated by an entrepreneur’s perception of possible failure (Bosma and Schutjens, 2011). The link between fear and failure has become extremely relevant in the entrepreneurial context where failure remains one of the most stigmatized outcomes (Shepherd and Haynie, 2011), but there are gender differences to the stigma of entrepreneurial failure due to socio-cultural factors. In a qualitative study by Shabbir and Gregorio (1996), a female entrepreneur noted that “if a man fails, people will sympathize; if a woman fails, people will laugh.” In addition, female entrepreneurs generally have a lower perception of the external environment than males (Shinnar et al., 2012), and show a greater fear of failure (Koellinger et al., 2013). Although entrepreneurship is considered a purposeful act (Morris et al., 2012), some women are forced to start their own businesses due to limited employment options or economic necessity. Fear of failure affects those who believe their options are limited more than those who believe they have other options (Atkinson, 1957).

We speculate that the dynamic of HmEP among female entrepreneurs is not influenced by fear of failure. Since female entrepreneurs who experience HmEP often participate in entrepreneurship because of inner motivation to pursue opportunities and independence, they can autonomously control their entrepreneurial activities and are less susceptible to failure syndrome (LeRouge et al., 2006). Moreover, female entrepreneurs who experience HmEP have intrinsic interest in their entrepreneurial activities and are able to pursue entrepreneurship in a flexible way (Vallerand et al., 2003; Vallerand, 2015). According to regulatory focus theory (Higgins, 1997, 1998), the pursuit of interest stimulates promotion focus motivation that leads to greater focus on positive results and favorable information, and positive information predicts an increase in positive affect (Casper et al., 2019). In a series of four experiments, Belanger et al. (2013) found that HmEP enables entrepreneurs to recognize and voluntarily participate in activities, thus reducing their focus on negative consequences related to failure and inhibiting the development of fear of failure. Therefore, we speculate that the dynamic of HmEP among female entrepreneurs is not affected by fear of failure.

Conversely, we speculate that the dynamic of ObEP in female entrepreneurs is influenced by fear of failure. Because female entrepreneurs who have ObEP are often forced to start a business due to involuntary unemployment or the pursuit of recognition by others, and they are controlled by their entrepreneurial activities (Vallerand et al., 2003; Vallerand, 2015). Based on regulatory focus theory (Higgins, 1997, 1998), when the motivation for participating in entrepreneurship stems from needs such as external protection and protection from harm, it stimulates prevention focus motivation. This drives individuals to respond to responsibility and safety issues, to dwell on negative outcomes and adverse information, and it predicts an increase in negative affect (Casper et al., 2019). Therefore, concerned that their entrepreneurial efforts will not be rewarded, these entrepreneurs are easily activated by failure-related signals. Furthermore, the self-worth of female entrepreneurs who have ObEP depends on achieving outstanding performance in the entrepreneurial realm (Lafrenière et al., 2011). They view entrepreneurial failure as personal failure, and thus they are overly sensitive to the threat of failure (Fox et al., 2002).

Prior studies have found that entrepreneurs’ fear of failure leads to one of two outcomes. On one hand, it may have a negative impact on entrepreneurial intention (Arenius and Minniti, 2005), opportunity recognition (Mitchell and Shepherd, 2010; Kollmann et al., 2017), and on re-enterprise (Hessels et al., 2011). Such an outcome leads to negative emotions and a pessimistic attitude about entrepreneurial activities. On the other hand, in order to avoid potential failure, some entrepreneurs engage more actively in entrepreneurial activities (Mitchell and Shepherd, 2012), search for novel solutions (McGrath, 2001), and become even more motivated to succeed (Cacciotti and Hayton, 2014).

Because individuals with ObEP are controlled by activity, their passion can lead to rigid adherence (Vallerand et al., 2003). Therefore, we speculate that female entrepreneurs who experience ObEP will dwell on the potential negative results of their efforts, thereby exacerbating their fear of failure. When experiencing high fear of failure, they are keenly attentive to any threatening stimuli and have difficulty separating their attention from the threat (Fox et al., 2002). Therefore, in order to avoid failure, they increase their passion toward entrepreneurial activities, resulting in rigid persistence behavior. This process becomes a cycle. Therefore, Hypothesis 3 is proposed.

*H3*: Fear of failure play a positive mediating role in the dynamics of ObEP among female entrepreneurs. That is, the dynamics of ObEP in female entrepreneurs is realized through entrepreneurial effort in the current period and fear of failure in the subsequent period.

## Materials and methods

### Sample

This study is based on a questionnaire survey distributed to female entrepreneurs at a college entrepreneurial training camp in Southwest China. We conducted the survey in three waves over a period of 5 months. In selecting the 5-month time frame, we relied on existing research designs on the dynamics of entrepreneurial passion (Cardon et al., 2013; Gielnik et al., 2015; Collewaert et al., 2016; Lex et al., 2020; Uy et al., 2021). Among them, Cardon et al. (2013) conducted a study on entrepreneurial passion over a period of 18 months and found that entrepreneurial passion was stable. Other longitudinal studies on entrepreneurial passion conducted over periods ranging from 3 weeks to 10 months found that entrepreneurial passion exhibits dynamic changes. Therefore, we deemed it feasible to investigate entrepreneurial passion changes in female entrepreneurs over a 5-month time span.

We distributed 110 questionnaires in the first wave; 105 valid questionnaires were returned. For the second wave, 98 valid questionnaires were recovered on the basis of the first valid questionnaire. For the third wave, 88 valid questionnaires were recovered on the basis of the second valid questionnaire. The total number of responses received was 88 × 3 = 264. Zhang and Willson (2006) suggest that the minimum sample size for a growth model should not be less than 50, and this sample size meets (exceeds) that standard.

In terms of educational background, 30.68% of our respondents had some or no college education; 51.14% had bachelor’s degrees, and 18.18% had master’s degrees or above. Entrepreneurial service businesses accounted for 62.50% and non-service businesses accounted for 37.50% of responses. The represented businesses ranged in fields including biotechnology, retail, manufacturing (e.g., food), services (e.g., law, consulting), and computers. The sample distribution was relatively extensive, lending authenticity to the questionnaire responses. Companies with less than 3 years of tenure accounted for 27.30%; companies with 3 to 8 years of tenure accounted for 55.68%; and companies with eight or more years of tenure accounted for 17.05% of responses. According to the 2019 GEM report, 41.7% of Chinese 18-to 64-year-olds identified opportunities but did not dare to start a business due to fear of failure. Clearly, fear of failure is common at various stages of entrepreneurial preparation and the entrepreneurial journey. The study of fear of failure in our sample is therefore plausible.

### Measures

In order to ensure the reliability and validity of our measurements, we quoted maturity scales that are accepted by most scholars with some modifications based on the actual situations of the female entrepreneurs in our study. We invited six entrepreneurs and three scholars in the field of entrepreneurial management to review and modify the final questionnaire. We employed Likert’s 7-point scoring scale to measure the degree of conformity between each item in the questionnaire and each entrepreneur’s actual situation, where 1 meant “strongly disagree” and 7 meant “strongly agree.” In order to quantify the variables, we used average values for all variables involved.

Entrepreneurial passion (T1, T2, T3). This study draws on the scale of Vallerand’s dualistic model of passion, with modifications based on related research (Ho and Pollack, 2014; Gielnik et al., 2015). We reduced the measurement of 12 items (6 each for harmony and obsessive passion) to 8 items (3 for HmEP and 5 for ObEP) to reduce the burden on respondents. The use of reduced scales in repeated measures is common because entrepreneurs are busy, and reduced scales eased the burden on these professionals (Foo et al., 2009; Gielnik et al., 2015; Uy et al., 2015; Murnieks et al., 2020). We also adjusted “this activity” in the scale to “entrepreneurship activity” with reference to the research design of Stroe et al. (2019). For HmEP, the scale consisted of three items, such as: “Entrepreneurial activity allows me to live memorable experiences.” For obsessive passion, the scale consisted of five items, such as: “I have a tough time controlling my need to do entrepreneurial activity.” Three waves of questionnaire surveys were distributed to participants in this study. The Cronbach’s α of the three waves of HmEP were 0.820, 0.838, and 0.813, and the Cronbach’s α of the three waves of ObEP were 0.916, 0.919, and 0.926, all of which are greater than 0.80. The KMO values of the three waves of HmEP were 0.672, 0.720, and 0.679, and the KMOs of the three waves of ObEP were 0.851, 0.855. 0.883, all of which are greater than 0.70. Therefore, both HmEP and ObEP values demonstrated satisfactory reliability and validity.

Entrepreneurial effort (T1, T2, T3) was assessed by using Foo et al. (2009) scale with proposed measurement items suitable for the Chinese context, such as “For the sake of the enterprise, I put a lot of energy into the management of the enterprise.” The Cronbach’s α of the three waves of entrepreneurship effort were 0.815, 0.824, and 0.869, all of which are greater than 0.80. The KMO values of the three waves of entrepreneurship effort were 0.671, 0.656, and 0.691, which are close to 0.70. Thus, entrepreneurial effort demonstrated satisfactory reliability and validity.

Fear of failure (T1, T2, T3). This variable was assessed using the fear of failure scale (PFAI-short; Conroy, 2001), modified to apply to entrepreneurship. The scale contains five items, such as “If I fail to start a business, I will worry about whether this proves my ability is not good.” The Cronbach’s α of the three waves of fear of failure were 0.846, 0.867, and 0.917, all of which are greater than 0.80. The KMO values of the three waves of fear of failure were 0.818, 0.821, and 0.822, all of which are close to 0.70. Thus, the reliability and validity of the fear of failure values are in line with the standard.

Control Variables. Existing literature shows that competitiveness and turbulence in an industry influence the performance of new ventures (Beckman et al., 2007; Brannon et al., 2013), which may affect entrepreneurial passion. In addition, based on experience, an entrepreneur’s education level and the age of the firm may affect the energy that an entrepreneur invests in entrepreneurial activities. Therefore, we selected: (1) the enterprise industry, (2) the education level of the entrepreneur, and (3) firm age as control variables. In order to limit the number of variables in our model, we re-assigned the three control variables and introduced them into the research model. We clustered the fields our sample entrepreneurs were involved in into two categories: (1) service industries, with an assigned value of 1, and (2) non-service industries, with an assigned value of 0. We classified the educational level of the entrepreneurs as: (1) less than a bachelor’s degree, with an assigned value of 0; (2) bachelor’s degree, with an assigned value of 1; and (3) higher than an undergraduate degree, with an assigned value of 2. We divided firm age into three categories: (1) less than 3 years, with an assigned value of 0; (2) 3 to 8 years, with an assigned value of 1; and (3) 8 years and above, with an assigned value of 2.

## Analysis and results

### Descriptive statistics and correlations

We used SPSS23 to analyze descriptive statistics and correlations for all variables used in this study, and the results are shown in Table 1. Descriptive statistical analysis revealed that: (1) the participants’ levels of HmEP and ObEP were unstable throughout the four time periods; (2) the standard deviation and variance were relatively large, indicating that the subjects’ HmEP and ObEP were discrete; and (3) the skewness and kurtosis were large, indicating that the participants’ HmEP and ObEP might not conform to a normal standard distribution. These results indicated that the participants’ entrepreneurial passion may be heterogeneous, so growth mixture modeling (GMM) was used to distinguish the different categories and the development trend of each category (Liu, 2007).

**Table 1:** Descriptive statistics and correlations.

	Variable	Mean	SD	Variance	Skewness	Kurtosis	1	2	3	4	5	6	7	8	9	10	11	12	13	14
1	HmEP1	6.496	0.663	0.439	−1.325	0.866														
2	HmEP 2	6.420	0.735	0.540	−1.255	1.107	0.490^**^													
3	HmEP 3	6.386	0.685	0.470	−1.088	1.474	0.341^**^	0.462^**^												
4	ObEP1	4.895	1.2883	1.660	−0.307	−0.461	0.371^**^	0.143	0.203											
5	ObEP 2	4.782	1.303	1.699	−0.498	−0.022	0.234^*^	0.291^**^	0.317^**^	0.575^**^										
6	ObEP 3	4.877	1.219	1.486	−0.128	−0.437	0.227^*^	0.257^*^	0.416^**^	0.406^**^	0.577^**^									
7	FF1	3.245	1.315	1.730	−0.132	−0.986	−0.016	−0.040	0.052	0.207	0.268^*^	0.256^*^								
8	FF2	3.575	1.307	1.707	−0.336	−0.421	0.176	0.021	0.097	0.349^**^	0.352^**^	0.363^**^	0.584^**^							
9	FF3	3.491	1.323	1.749	−0.110	−0.732	0.195	0.048	0.172	0.334^**^	0.295^**^	0.402^**^	0.611^**^	0.707^**^						
10	EE1	5.723	1.0236	1.048	−0.657	0.268	0.391^**^	0.389^**^	0.300^**^	0.378^**^	0.270^*^	0.291^**^	0.030	0.175	0.179					
11	EE2	5.587	0.975	0.950	−0.470	−0.055	0.299^**^	0.475^**^	0.442^**^	0.259^*^	0.302^**^	0.310^**^	−0.021	0.061	0.197	0.615^**^				
12	EE3	5.489	1.0768	1.160	−0.166	−0.942	0.258^*^	0.354^**^	0.613^**^	0.181	0.111	0.332^**^	−0.041	0.069	0.092	0.436^**^	0.589^**^			
13	Education	0.875	0.692	0.478	0.170	−0.875	−0.005	−0.106	−0.220^*^	−0.203	−0.247^*^	−0.201	−0.193	−0.189	−0.244^*^	−0.120	−0.129	−0.123		
14	Industry	0.625	0.487	0.237	−0.525	−1.765	0.073	−0.058	0.003	0.043	−0.072	0.073	−0.099	0.018	−0.021	−0.026	−0.096	−0.012	0.166	
15	Firm age	0.898	0.662	0.438	0.113	−0.680	0.003	−0.029	−0.140	0.009	−0.021	−0.061	−0.032	−0.038	0.092	−0.003	−0.066	−0.020	−0.279^**^	−0.227^*^

N = 88. HmEP = Harmonious Entrepreneurial Passion; ObEP = Obsessive Entrepreneurial Passion; FF = Fear of Failure; EE = Entrepreneurial Effort.

* *p* < 0.05;

** *p* < 0.01.

### Hypothetical test

We used Mplus8.3 to analyze the latent growth model over the three periods of entrepreneurial passion (HmEP and ObEP). Enterprise industry, the entrepreneur’s education level, and firm age were used as control variables. The three periods of entrepreneurial passion were used as dependent variables. This study is a free estimation time parameter model (Wang et al., 2014). Since the number of potential categories and whether there was variation within the categories was unknown, we compared GMMs of 4 categories of HmEP and ObEP of the female entrepreneurs tested, respectively (Tables 2, 3).

**Table 2:** Comparison of fit degree of latent variable growth models of different classifications of HmEP.

Model	DF	LMR-LRT *p*	BLRT *p*	AIC	BIC	aBIC	Entropy
GMM-1C	9	–	–	525.153	547.449	519.048	–
GMM-2C	**12**	**0.030**	**0.000**	**495.928**	**525.656**	**487.789**	**0.936**
GMM-3C	15	0.040	0.000	450.151	487.311	439.977	0.976
GMM-4C	18	0.134	0.000	444.109	488.701	431.901	0.967
Add control variables to the HmEP GMM-2C model							
Industry	14	0.026	0.000	497.156	531.838	487.660	0.935
Education	14	0.069	0.000	495.028	529.710	485.532	0.935
Firm age	14	0.046	0.000	499.848	534.531	490.352	0.936

DF= Number of Free Parameters; LMR-LRT= Lo-Mendell-Rubin Adjusted LRT Test; BLRT= Parametric Boostrapped Likelihood Ratio; AIC= Akaike; BIC= Bayesian; aBIC= Adjusted BIC; Entropy= Entropy.

**Table 3:** Comparison of fit degree of latent variable growth models of different classifications of ObEP.

Model	DF	LMR-LRT *p*	BLRT *p*	AIC	BIC	aBIC	Entropy
GMM-1C	9			818.354	840.650	812.250	
GMM-2C	**12**	**0.234**	**0.032**	**810.442**	**840.170**	**802.303**	**0.833**
GMM-3C	15	0.658	0.697	814.589	851.749	804.415	0.783
GMM-4C	18	0.240	0.156	810.413	855.005	798.204	0.890
Add control variables to the ObEP GMM-2C model							
Industry	14	0.256	0.042	810.749	845.431	801.253	0.855
Education	14	0.437	0.096	808.729	843.412	799.234	0.837
Firm age	14	0.277	0.048	814.283	848.966	804.787	0.834

DF= Number of Free Parameters; LMR-LRT= Lo-Mendell-Rubin Adjusted LRT Test; BLRT= Parametric Boostrapped Likelihood Ratio; AIC= Akaike; BIC= Bayesian; aBIC= Adjusted BIC; Entropy= Entropy.

#### HmEP

We chose the GMM-2C model as the best fitting model because, compared to the other three category models, this model has a significant value of p of BLRT, a smaller BIC, AIC = 495.928, aBIC = 487.789, Entropy = 0.936, both of which meet the fitting standard (Nylund et al., 2007). The value of p of the LMR taking the 3-class model increased from 0.030 to 0.040, making the LMR more inclined to select the two-class model. The GMM-2C model divides the HmEP development trend into two subgroups. According to the parameter estimation of the model, the proportion of entrepreneurs in the first subgroup is the largest, at about 90%. The intercept mean for these entrepreneurs’ HmEP is 6.685 (SE = 0.054, *t* = 124.752, *p* = 0.000 < 0.01), and the slope mean is-0.135 (SE = 0.073, *t* = −1.849, *p* = 0.065 < 0.1). This subgroup of entrepreneurs is the most common type. They initially have high HmEP, but then, in order to maintain work-family balance, they transfer some of their passion to family. Thus HmEP gradually weakens, showing a downward trend. The proportion of entrepreneurs in the second subgroup is about 10%. The intercept mean for these entrepreneurs’ HmEP is 5.091 (SE = 0.188, *t* = 27.030, *p* = 0.000 < 0.01), and the slope mean is 0.527 (SE = 0.297, *t* = 1.772, *p* = 0.076 < 0.1). These entrepreneurs initially have low HmEP, but as the entrepreneurial process progresses, their HmEP gradually rises. The variance of the intercept factor of HmEP is 0.048 (SE = 0.063, *t* = 0.758, *p* = 0.448 > 0.1), and the variance of the slope factor is 0.033 (SE = 0.043, *t* = 0.767, *p* = 0.443 > 0.1). This shows that within each subgroup, there are no significant differences among individuals in the initial state of HmEP and the growth rate. The classification probabilities for the most likely latent class membership (column) by latent class (row) in the two categories of harmonious passion are 97.90 and 99.7%, indicating that the results of the two latent category classification models are credible. This finding provides support for H1a. It can be seen from Table 2 and the estimated data that the model with control variables is not significantly different from the original model.

#### ObEP

We chose the GMM-2C model as the best fitting model because, compared to the other three category models, it has a significant value of p of BLRT, a smaller BIC, AIC = 810.442, aBIC = 802.303, Entropy = 0.833, both of which meet the fitting standard (Nylund et al., 2007). The GMM-2C model divides the ObEP development trend into two sub-categories. The first category accounted for about 84% of the entrepreneurs. The intercept mean of the ObEP for this category of entrepreneurs is 4.907 (SE = 0.133, *t* = 36.820, *p* = 0.000 < 0.01), and the slope mean is 0.209 (SE = 0.109, *t* = 1.905, *p* = 0.057 < 0.1). This subgroup has high initial ObEP, and their ObEP has a significant upward trend over time. Entrepreneurs in this subgroup belong to the normal population. They are dominated by entrepreneurial activities, so the ObEP of entrepreneurial activities increases over time. The second category accounted for about 16% of the entrepreneurs. The intercept mean for this category of entrepreneurs ObEP is 4.743 (SE = 0.418, *t* = 11.358, *p* = 0.000 < 0.01), and the slope mean is -1.545 (SE = 0.359, *t* = −4.306, *p* = 0.000 < 0.1). This subgroup has low initial ObEP, indicating that they are less dominated by entrepreneurial activities. Therefore, as the entrepreneurial process advances, the external pressures are also low and they may relinquish control of their entrepreneurial activities. Thus, their ObEP becomes weaker and weaker. The variance of the intercept factor of ObEP is 0.662 (SE = 0.197, *t* = 3.366, *p* = 0.001 < 0.01), and the variance of the slope factor is-0.366 (SE = 1.491, *t* = −0.245, *p* = 0.806 > 0.1). This shows that within each subgroup, there are significant variations among individuals in the initial state of ObEP, but there is no significant difference in the growth rate. The classification probabilities for the most likely latent class membership (column) by latent class (row) in the two categories of obsessive passion are 90.10 and 96.40%, indicating that the results of the two latent category classification models are credible. This finding provides support for H1b. It can be seen from Table 3 and the estimated data that the model with control variables is not significantly different from the original model.

**Figure 1:**
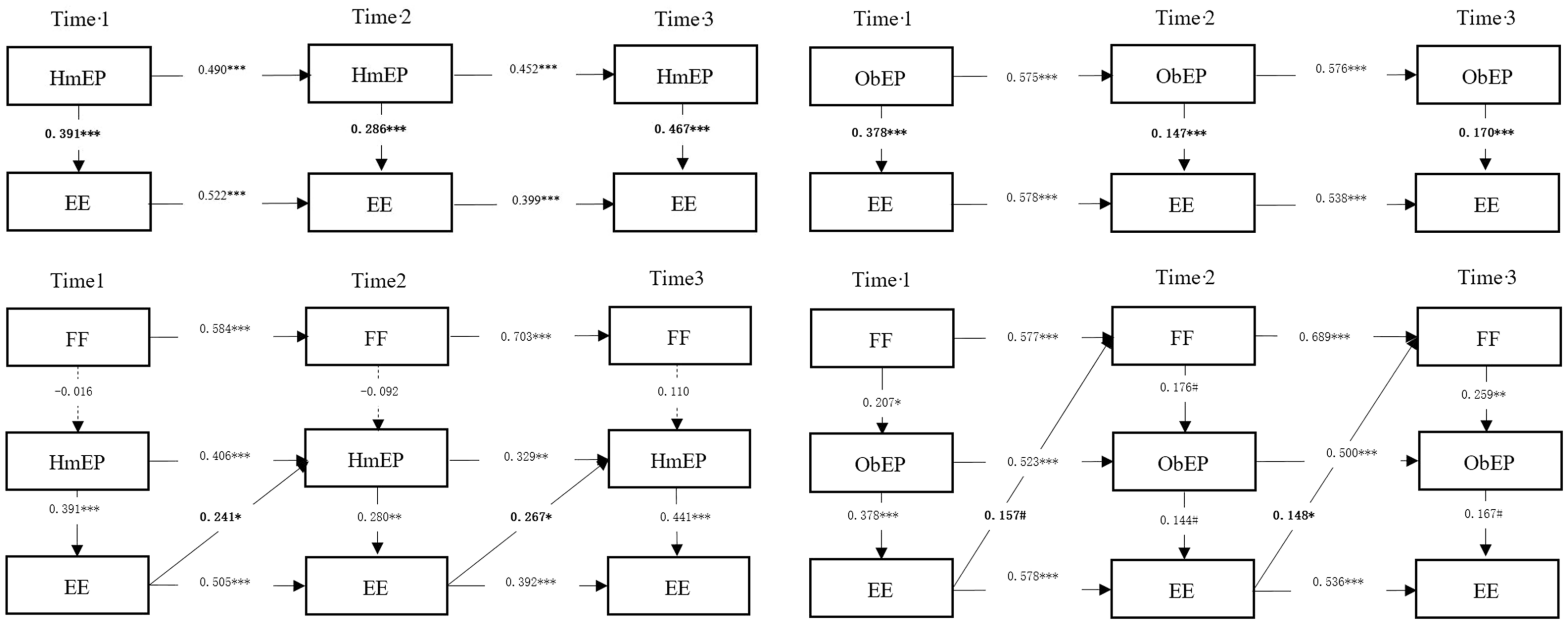
Standardized estimates for significant paths in the four models.

To test H2 and H3, we adopted a cross-lag analysis on our data using MPlus8.3 (Newman, 2014) and fit four path models to our data (see Figure 1): HmEP-EE base model (M1), ObEP-EE basic model (M2), HmEP-EE-FF dynamic model (M3), and ObEP-EE-FF dynamic model (M4). The M1 and M2 base models included only temporal stabilities (i.e., the sequentially affected paths at 3 time points per variable) and synchronous effects (i.e., the effect of HmEP/ObEP on EE at the same time). The HmEP-EE-FF dynamic model (M3) is based on the base model M1 and adds the FF of the same period, then adds the lag path of EE to the HmEP of the later period. The ObEP-EE-FF dynamic model (M4) is based on the basic model M2 and adds the FF of the same period, then adds the lag path of EE to the FF of the later period.

We assessed the fit of the four models. Table 4 shows the fit indices of the four models and the results of the chi-square difference tests. First, the HmEP-EE base model (M1) included temporal stabilities as well as synchronization effects and had a good fit, χ^2^(15) = 187.679, *p* < 0.001, CFI = 0.951, TLI = 0.909, RMSEA = 0.109, SRMR = 0.116. Stability coefficient estimates for HmEP were 0.490 (SE = 0.081, *t* = 6.041, *p* = 0.000 < 0.001) and 0.452 (SE = 0.085, *t* = 5.332, *p* = 0.000 < 0.001). The estimated stability coefficients of EE are 0.522 (SE = 0.079, *t* = 6.569, *p* = 0.000 < 0.001) and 0.399 (SE = 0.085, *t* = 4.700, *p* = 0.000 < 0.001), indicating that there is an auto-regression between HmEP and EE effect. The estimated coefficients of the synchronization effect of HmEP-EE at three time points were 0.391 (SE = 0.090, *t* = 4.329, *p* = 0.000 < 0.001) and 0.286 (SE = 0.089, *t* = 3.216, *p* = 0.001 < 0.005) and 0.467 (SE = 0.084, *t* = 5.586, *p* = 0.000 < 0.001; all coefficient estimates given are standardized), indicating that the HmEP of female entrepreneurs increased their entrepreneurial effort for that time period.

**Table 4:** Fit indices and chi-square test results for the four models.

Model	χ^2^	df	CFI	TLI	RMSEA	SRMR
HmEP-EE base model (M1)	187.679^***^	15	0.951	0.909	0.109	0.116
ObEP-EE base model (M2)	179.560^***^	15	0.986	0.973	0.058	0.064
HmEP-EE-FF dynamic model (M3)	313.686^***^	36	0.971	0.952	0.065	0.076
ObEP-EE-FF dynamic model (M4)	321.532^***^	36	0.963	0.939	0.074	0.069

CFI = comparative fit index; TLI = Tucker Lewis index; RMSEA = root mean square error of approximation; SRMR = standardized root mean square residual.

*** *p* < 0.001.

The ObEP-EE base model (M2) included temporal stabilities as well as synchronization effects and had a good fit, χ^2^(15) = 179.560, *p* < 0.001, CFI = 0.986, TLI = 0.973, RMSEA = 0.058, SRMR = 0.064. Stability coefficient estimates for ObEP were 0.575 (SE = 0.071, *t* = 8.056, *p* = 0.000 < 0.001) and 0.576 (SE = 0.071, *t* = 8.096, *p* = 0.000 < 0.001). Stability coefficient estimates for EE were 0.578 (SE = 0.072, *t* = 8.037, *p* = 0.000 < 0.001) and 0.538 (SE = 0.078, *t* = 6.905, *p* = 0.000 < 0.001). The estimated coefficients of the synchronization effect of ObEP-EE at three time points were 0.378 (SE = 0.091, *t* = 4.140, *p* = 0.000 < 0.001) and 0.147 (SE = 0.086, *t* = 1.702, *p* = 0.089 < 0.1) and 0.170 (SE = 0.091, *t* = 1.864, *p* = 0.062 < 0.1), indicating that the ObEP of female entrepreneurs increased their entrepreneurial effort for that time period.

Second, the HmEP-EE-FF dynamic model (M3) includes the base model M1 and adds the FF of the same time, as well as the lag path of EE to the later time of HmEP. The model had a good fit, χ^2^(36) = 313.686, *p* < 0.001, CFI = 0.971, TLI = 0.952, RMSEA = 0.065, SRMR = 0.076. Based on the model results, we found that both the temporal stability and synchronization effects of HmEP-EE were significant (*p* < 0.001). The time stability of FF was significant (*p* < 0.001), but the synchronization effect of FF-HmEP was not significant (*p* > 0.1), indicating that the current HmEP of female entrepreneurs was not affected by their current fear of failure. The lag path coefficients of EE to HmEP in the later time period are 0.242 (SE = 0.095, *t* = 2.561, *p* = 0.010 < 0.05) and 0.267 (SE = 0.104, *t* = 2.578, *p* = 0.010 < 0.05), meaning that the entrepreneurial effort of female entrepreneurs had a significant positive lagging effect on the HmEP for the subsequent time period. The results of M1 and M3 support Hypothesis 2a. That is, entrepreneurial effort plays a mediating role in the dynamics of HmEP of female entrepreneurs.

Finally, the ObEP-EE-FF dynamic model (M4) includes the base model M2 and adds the FF of the same time and the lag path of EE to the FF of the later time period. The model had a good fit, χ^2^(36) = 321.532, *p* < 0.001, CFI = 0.963, TLI = 0.939, RMSEA = 0.074, SRMR = 0.069. Model results showed that both ObEP-EE temporal stability and synchronization effects were significant (*p* < 0.001). The time stability of FF was significant (*p* < 0.001), and the synchronization effect of FF-ObEP was significant, with coefficients of 0.207 (SE = 0.102, *t* = 2.027, *p* = 0.043 < 0.05), 0.176 (SE = 0.093, *t* = 1.879, *p* = 0.061 < 0.1) and 0.259 (SE = 0.088, *t* = 2.943, *p* = 0.003 < 0.01), indicating that the current ObEP of female entrepreneurs was indeed positively affected by fear of failure. In addition, the lag path coefficients of EE for the later time of FF were 0.157 (SE = 0.084, *t* = 1.879, *p* = 0.060 < 0.1) and 0.148 (SE = 0.072, *t* = 2.049, *p* = 0.040 < 0.05), meaning that female entrepreneurs who experienced ObEP were indeed affected by negative information and had a fear of failure after putting in effort, which is consistent with our hypothesis. Overall, the results of M2 and M3 support Hypotheses 2b and Hypotheses 3. That is, entrepreneurial effort and fear of failure play a mediating role in the dynamics of ObEP of female entrepreneurs. Specifically, the dynamics of ObEP of female entrepreneurs is achieved through the entrepreneurial efforts of the current period and the fear of failure in subsequent period.

## Discussion

We focused our research on the entrepreneurial passion of female entrepreneurs, endeavored to analyze the dynamic changes in their entrepreneurial passion over time, and explored the internal mechanism underpinning the dynamic. Specifically, using a 5-month, three-wave longitudinal survey of 88 female entrepreneurs, we employed the latent variable growth model and a cross-lag model and examined (1) the dynamic trends of female entrepreneurs’ HmEP and ObEP, and (2) the differing roles of female entrepreneurs’ entrepreneurial effort and fear of failure in the dynamic evolution of the two types of entrepreneurial passion. Next, we discuss the theoretical implications, practical implications, and limitations of this study as well as possible future research directions.

### Theoretical implications

Our study revealed that the entrepreneurial passion of female entrepreneurs is indeed dynamic and the dynamic trends of HmEP and ObEP are different. More interestingly, the factors that affect the temporal dynamics of female entrepreneurs’ HmEP and ObEP differ. Specifically, female entrepreneurs’ entrepreneurial effort has a stimulating effect on subsequent changes of HmEP, while entrepreneurial effort has a stimulating effect on subsequent changes of ObEP by affecting fear of failure. This study expands the body of knowledge on the dynamics of entrepreneurial passion and offers novel research constructs on the entrepreneurial passion of female entrepreneurs.

First, we enhance the corpus of literature on female entrepreneurship with a female-centered entrepreneurship study. Often when we refer to successful entrepreneurs, we think of men (e.g., Bill Gates, Steve Jobs, Mark Zuckerberg, Jack Ma, etc.), and the early literatures mainly describe entrepreneurs as “male” rather than “female” (Bird and Brush, 2002; Gupta et al., 2008), while ignoring the heterogeneity of female entrepreneurs. To move beyond male-centric interpretations of entrepreneurship, we focused on the entrepreneurial passion trends of female entrepreneurs. Passion differs between men and women (Sigmundsson et al., 2020). Murnieks et al. (2020) found that men and women do not develop entrepreneurial passion in exactly the same way and called for more work to study the gendered nature of entrepreneurial passion. This study answers that call and explores the inner dynamics of entrepreneurial passion among female entrepreneurs. Our findings help explain why some female entrepreneurs are more likely than others to succeed or to abandon entrepreneurship.

Second, based on the two motivations of female entrepreneurs, active or forced to participate in entrepreneurship (Jafari-Sadeghi, 2020), we chose the DPM of Vallerand et al. (2003) and used HmEP and ObEP to describe the dynamic evolution of female entrepreneurial passion, thus providing unique theoretical support for entrepreneurial research focused on female entrepreneurs. Our study found that female entrepreneurs’ HmEP and ObEP both show temporal dynamics, but the development trends of the two are heterogeneous. HmEP has two development trend subgroups: one in which initial harmonious passion is high but then trends downward and another in which initial harmonious passion is low but then trends upward. That is to say: over time, too much or too little HmEP invested in entrepreneurial activities forces female entrepreneurs to balance the energy invested in entrepreneurial activities so that family and work occupy a more stable state. By contrast, two different development trends are observed in those with obsessive passion: a subgroup in which initial obsessive passion is high and then trends upward and another in which initial obsessive passion is low and trends further downward over time. That is to say: female entrepreneurs with high ObEP devote increased energy to entrepreneurial activities over time because they are controlled by entrepreneurial activities (Vallerand et al., 2003). However, female entrepreneurs with low ObEP gradually relinquish control of entrepreneurial activities resulting in a gradual decrease in ObEP. These women may even withdraw from entrepreneurial activities altogether (Justo et al., 2015). The conclusions of this study confirm the entrepreneurial passion of female entrepreneurs is indeed dynamic and the dynamic trends of HmEP and ObEP are different. They also raise questions about the conclusions of previous studies based on the premise that passion is a stable emotion and provide a new direction for future research on entrepreneurial passion.

Third, by examining the differing roles of female entrepreneurs’ entrepreneurial effort and fear of failure in the dynamic evolution of the two types of entrepreneurial passion, we further enrich the research corpus on the dynamics of female entrepreneurs’ entrepreneurial passion. Since HmEP and ObEP are distinct structures, it is not surprising that they are affected along different pathways (Mageau et al., 2009; Vallerand, 2015). This study found that the entrepreneurial effort of female entrepreneurs played an important role in the dynamic changes of HmEP, while entrepreneurial effort and fear of failure both played important roles in the dynamic changes of ObEP. Specifically, after female entrepreneurs’ HmEP stimulated entrepreneurial effort, they focused more attention to positive information, thereby increasing their HmEP in the subsequent period. After female entrepreneurs’ ObEP stimulated entrepreneurial effort, they tended to dwell on negative information, thereby increasing the fear of failure in the subsequent period. In order to avoid failure, they were compelled to devote more passion to their entrepreneurial activities. This conclusion reveals the internal mechanism of the dynamic evolution of HmEP and ObEP from the perspective of female entrepreneurs and emphasizes the stimulating role of entrepreneurial effort and fear of failure in the evolution of entrepreneurial passion. In particular, it emphasizes the relationship between entrepreneurial effort and HmEP changes and the relationship between entrepreneurial effort, fear of failure, and ObEP changes.

At the same time, this conclusion extends the literature on entrepreneurial effort and fear of failure. Our research partially corroborates the findings of Gielnik et al. (2015) that entrepreneurs’ entrepreneurial efforts have a positive impact on entrepreneurial passion. It is worth noting that their research uses the definition of entrepreneurial passion by Cardon et al. (2009) to study the relationship between entrepreneurial effort and entrepreneurial passion. However, this study focuses on the existence of two types of motivation for women to actively and passively participate in entrepreneurship (Jafari-Sadeghi, 2020) and employs a DPM (Vallerand et al., 2003) that better matches the two types of motivation. An analysis of the dynamics of entrepreneurial effort with HmEP and ObEP extends the work of Gielnik et al. (2015).

In most previous studies, fear of failure was considered to be an impeding factor for entrepreneurs (Arenius and Minniti, 2005), but this has limited understanding of how entrepreneurs experience fear of failure during the entrepreneurial process. We found that female entrepreneurs’ fear of failure significantly contributed to the dynamic evolution of ObEP. Our conclusions help scholars, entrepreneurs, and others view fear of failure more comprehensively and accurately.

### Practical implications

Although women make up about half of the world’s population, they are still underrepresented among entrepreneurs (Xie and Wu, 2021). Socio-cultural resource constraints and patriarchal structures limit women’s participation in entrepreneurship (De Clercq and Brieger, 2021; Xie and Wu, 2021). Given that female entrepreneurship provides opportunities for individual and social growth (Mari et al., 2016), research on female entrepreneurship can contribute to the development of society. The dynamics of entrepreneurial passion can describe in detail the positive emotional changes of entrepreneurs who continue to invest in innovation and entrepreneurial activities. Therefore, this research uses the temporal dynamics of the dualistic model of passion as a starting point to explore the inner impact of female entrepreneurs’ entrepreneurial effort and fear of failure on the temporal dynamics of entrepreneurial passion, which is of great significance for management practice.

Implications for female entrepreneurs: (1) the temporal dynamics of entrepreneurial passion objectively exist. Entrepreneurial passion has a significant impact on entrepreneurial performance (Cardon et al., 2009), and the temporal dynamics of entrepreneurial passion make entrepreneurial performance unstable. Therefore, female entrepreneurs should be aware of differences in motivation when pursuing entrepreneurial careers, recognize the follow-up effects of different types of entrepreneurial passion, and adjust their levels of passion in the entrepreneurial process according to the needs of their specific entrepreneurial situations. This will help stabilize changes in entrepreneurial performance and increase the probability of entrepreneurial success. (2) Entrepreneurial effort and fear of failure have differing effects on the two types of entrepreneurial passion of female entrepreneurs. On the one hand, entrepreneurial effort can enhance progress and enterprise development (Isen and Labroo, 2003) and inspire the entrepreneur’s subsequent HmEP during the entrepreneurial process. This then becomes a virtuous cycle leading, eventually, to entrepreneurial success. On the other hand, since female entrepreneurs are generally more risk-sensitive than males (Dohmen et al., 2011), female entrepreneurs should recognize that fear of failure may not only engender negative emotions (Cacciotti et al., 2016) but may also affect ObEP later in the entrepreneurial process and compel them to become more actively involved in entrepreneurial activities.

Implications for society: Female entrepreneurs have various motivations for participating in entrepreneurship. Distinguishing and understanding different types of entrepreneurial motives can help explain the temporal dynamics of entrepreneurial passion that objectively exist and enable a better understanding of women’s entrepreneurial experiences over time. This may lead to a more conducive entrepreneurial environment for female entrepreneurs and aid overall social development (Mari et al., 2016; Xie et al., 2021). In addition, since entrepreneurial effort and fear of failure impact the dynamics of female entrepreneurs’ entrepreneurial passion, governments, NGOs or other organizations should consider providing entrepreneurial training for female entrepreneurs. As women learn how to use their time and energy more effectively and come to better understand the positive effect of fear of failure, they can maintain greater and more consistent passion for their entrepreneurial activities.

### Limitations

As with any research project, this study has some limitations. First, this research focuses only on the temporal dynamics of female entrepreneurs’ HmEP and ObEP. Although it expands the body of knowledge on female entrepreneurship, it does not address differences between male and female entrepreneurs’ entrepreneurial passion development trends. Indeed, we are unable to fully demonstrate the differing entrepreneurial characteristics of men and women, and therefore our results may not be generalizable to male entrepreneurs. Second, we analyzed various previous research designs on the dynamics of entrepreneurial passion (Cardon et al., 2013; Gielnik et al., 2015; Collewaert et al., 2016; Lex et al., 2020; Uy et al., 2021), and it should be noted Cardon et al. (2013) found entrepreneurial passion to be stable over time. This is probably because their study spanned 18 months. Other studies, which spanned 3 weeks to 10 months, found entrepreneurial passion to be dynamic. We determined that a five-month time frame would appropriately capture the dynamics of entrepreneurial passion among female entrepreneurs, and our hypothesized relationship was supported. After this time frame, however, it is less clear whether these effects continue.

### Future research directions

Given our study’s limitations, we highlight several avenues for further research. First, scholars may evaluate the relationships analyzed in this study among male entrepreneurs to further clarify the varying traits of men and women in the entrepreneurial field. Also, because gender roles may vary by culture (Jones and Tullous, 2002; Baughn et al., 2006; Jaim, 2020), future researchers can evaluate entrepreneurs in countries other than China. Second, we found that female entrepreneurs had a downward trend in high HmEP and an upward trend in low HmEP over the five-month time frame. Future researchers could look at longer time periods to identify longer-term fluctuations in entrepreneurial passion. Third, we have proposed a classification of differing entrepreneurial passions and found heterogeneity and instability in female entrepreneurs’ HmEP and ObEP. However, the factors that led to this classification are not discussed or verified. Future research may expand on this aspect. In addition, further research on the temporal dynamics of entrepreneurs’ HmEP and ObEP, whether by enriching the independent and dependent variables, exploring the dynamics of HmEP and ObEP over a longer time period, or exploring a possible conversion between HmEP and ObEP, would be worthwhile.

## Data availability statement

The original contributions presented in the study are included in the article/supplementary material; further inquiries can be directed to the corresponding author.

## Author contributions

YR was in charge of investigation, methodology, writing—original draft, and editing. XF and QX were in charge of conceptualization, methodology, writing—review and editing, and funding acquisition. TC was in charge of literature review, investigation, methodology—original draft. All authors contributed to the article and approved the submitted version.

## Funding

This work was supported by the National Natural Science Foundation of China (71672150).

## Conflict of interest

The authors declare that the research was conducted in the absence of any commercial or financial relationships that could be construed as a potential conflict of interest.

## Publisher’s note

All claims expressed in this article are solely those of the authors and do not necessarily represent those of their affiliated organizations, or those of the publisher, the editors and the reviewers. Any product that may be evaluated in this article, or claim that may be made by its manufacturer, is not guaranteed or endorsed by the publisher.
